# Genetic and Biotechnological Approaches to Gestational Diabetes Mellitus: Advancing Diagnostics, Treatment Strategies, and Public Health Implications

**DOI:** 10.7759/cureus.70386

**Published:** 2024-09-28

**Authors:** Shahzeb Leghari, Raziel Santos, Abdullah Ghumman, Saira Khan, Muhammad Shoaib, Sana Noor, Arsalan Rasheed

**Affiliations:** 1 Medical School, St. George’s University, Brooklyn, USA; 2 Medical School, St. George’s University, San Jose, USA; 3 Medicine and Surgery, King Edward Medical University, Lahore, PAK; 4 Gynecology, Khyber Teaching Hospital, Peshawar, PAK; 5 Biochemistry, Gajju Khan Medical College, Swabi, PAK; 6 Community Medicine, Avicenna Medical and Dental College and Hospital, Lahore, PAK; 7 Molecular Biology and Genetics, Abdul Wali Khan University Mardan, Mardan, PAK

**Keywords:** fto, genetic markers, gestational diabetes mellitus, lifestyle factors, personalized medicine, tcf7l2

## Abstract

Background

Gestational diabetes mellitus (GDM) is a disorder where pregnant women have difficulty processing glucose, which influences the mother’s and the fetus’s health. The rising prevalence of GDM, often linked to obesity, highlights the need to study its processes and develop appropriate treatment techniques. Conventional diagnostic approaches may not accurately anticipate or address genetic predispositions, emphasizing the need for further investigation.

Objective

This study aims to explore genetic and biotechnological approaches to improve diagnostics, treatment, and public health strategies for GDM.

Methodology

This research was carried out in two hospitals in Pakistan over a period of 12 months, from January to December 2023. A sample size of 260 was determined using an anticipated GDM prevalence of 15%. The data were obtained by administering structured questionnaires, doing anthropometric measures, and analyzing blood samples for genetic information. The statistical study included the use of descriptive statistics, chi-square tests, and logistic regression to examine the relationships between genetic markers and the risk of GDM. The significance level of these associations was assessed using a p-value of <0.05.

Results

Among the 260 individuals with GDM included in the research, 52 (20.00%) and 73 (28.08%) patients had the TCF7L2 risk variation. The study found that the TCF7L2 and FTO risk polymorphisms were linked to a higher likelihood of developing GDM among the participants. The association was statistically significant, with p-values of 0.012 and 0.045, respectively. Logistic regression analysis showed that the TCF7L2 risk variant had an odds ratio of 2.35 (95% confidence interval [CI]: 1.21-4.56, p = 0.012), while the FTO risk variant had an odds ratio of 1.97 (95% CI: 1.05-3.70, p = 0.045). There is a substantial association between consuming a large amount of sugar (OR: 2.10, 95% CI: 1.15-3.82, p = 0.017) and engaging in little physical activity (OR: 1.85, 95% CI: 1.07-3.22, p = 0.025) and an increased risk of GDM. Treatment brought the glucose levels of people with GDM down to normal in 68.49% of cases.

Conclusion

Integrating genetic screening for TCF7L2 and FTO variants with lifestyle modifications may enhance early detection and personalized management of GDM, leading to improved maternal and fetal health outcomes.

## Introduction

Gestational diabetes mellitus (GDM) is a medical disorder where there is a reduced ability to tolerate glucose during pregnancy, which adversely affects the mother’s and the fetus’s health [1,2]. The growing occurrence of GDM, along with the escalating rates of obesity, requires an immediate investigation into its underlying processes and efficient ways for its control [3]. The current diagnostic methods mostly depend on glucose tolerance tests, which, while well-established, often lack the ability to accurately anticipate GDM in its first phases and may disregard the genetic predispositions that play a role in its development [4].

Recent breakthroughs in genetic studies and technologies provide significant opportunities for improving our knowledge of GDM [5]. Polymorphisms in insulin signaling pathways and glucose metabolism genes have been identified as genetic variables that have a role in the development of GDM [6]. Nevertheless, the present research does not sufficiently address the complex relationship between genetic predispositions and environmental influences [7]. Biotechnological advancements, such as biomarkers and sophisticated imaging methods, provide prospective means for detecting and monitoring GDM at an early stage. However, their practical use in clinical settings is still in its early phases [8].

Concurrently, the main emphasis in treating GDM is on making changes to one’s lifestyle and using insulin therapy. There is an increasing interest in the potential of nutraceuticals and customized medicine in managing this condition [9]. Although some studies indicate that certain dietary elements may reduce the incidence of GDM, there is a lack of comprehensive guidelines that incorporate genetic knowledge into dietary recommendations [10,11]. In addition, the potential of tailored therapeutic treatments using biotechnological advancements has not been thoroughly investigated, resulting in a notable deficiency in existing treatment strategies [12].

Although the genetic and biotechnological sectors have made significant progress, there is still a lack of a comprehensive framework that connects genetic predisposition with advanced diagnostic and treatment approaches for GDM. It is crucial to address this gap in order to create more efficient and tailored treatment choices that have the potential to greatly enhance the outcomes for both mothers and their unborn babies. The objective of this study was to investigate the genetic factors, specifically focusing on polymorphisms in the TCF7L2 and FTO genes, that influence the risk of GDM. Additionally, the study aimed to evaluate biotechnological approaches that can enhance diagnostics, treatment, and public health strategies for managing GDM, with a particular emphasis on integrating genetic screening into routine clinical practice.

## Materials and methods

Study design and settings

This research employed a cross-sectional design and was conducted in the Khyber Teaching Hospital, Peshawar, Pakistan, and Avicenna Hospital, Lahore, Pakistan, over 12 months from January 2023 to December 2023. These hospitals provide advanced treatment and serve a diverse patient population, making it an ideal setting for studying GDM and its related genetic and biotechnological aspects to analyze the public health strategies for managing and preventing GDM, considering factors such as community health education, accessibility of prenatal care, and the integration of genetic screening into public health policies.

Inclusion and exclusion criteria

Inclusion criteria included pregnant women aged 18-44 years who sought standard prenatal care at the hospital. The exclusion criteria included women with pre-existing diabetes, women who had been diagnosed with GDM in prior pregnancies, women with multiple gestations, and women with any chronic conditions that might potentially disrupt glucose metabolism (such as severe hypertension or renal impairment).

Sample size

The sample size was determined using the formula for calculating sample size for a proportion in a cross-sectional study:

𝑛 = 𝑍^2^ × 𝑝 × (1 − 𝑝)/𝑑^2^

where 𝑛 = required sample size; 𝑍 = 𝑍-value (the standard normal variate corresponding to the desired confidence level, typically 1.96 for a 95% confidence level); 𝑝 = estimated prevalence of GDM (assumed to be 15% or 0.15); 𝑑 = margin of error (desired precision, set at 5% or 0.05).

Substituting the values:

𝑛 = 1.96^2^ × 0.15 × (1 − 0.15)/0.05^2^

𝑛 = 3.8416 × 0.15 × 0.85/0.0025

𝑛 = 3.8416 × 0.1275/0.0025

𝑛 = 0.490056/0.0025

𝑛 = 196.02.

The calculated sample size is approximately 196 participants. To account for potential non-responses or dropouts, an additional 25% was added:

Adjusted sample size = 196 + (0.25 × 196) = 245.

Rounding up to ensure sufficient power and to accommodate variability, the final sample size was set at 260 participants. This sample size is expected to provide adequate statistical power to identify significant associations between genetic predispositions, lifestyle factors, and GDM risk, as well as to evaluate the effectiveness of public health interventions. This calculation resulted in a necessary sample size of 260 participants, ensuring sufficient statistical power to identify significant relationships between genetic predispositions, lifestyle factors, and GDM risk, as well as the potential impact of public health interventions.

Data collection and laboratory procedures

Data collection involved administering a structured questionnaire that gathered demographic details, medical history, lifestyle factors (e.g., nutrition and physical activity), and family history of diabetes. Anthropometric measurements, including body mass index (BMI) and waist circumference, were recorded.

Blood sample collection and handling, DNA extraction, visualization, purification, and quality control were performed using standard protocols as previously described by Habib et al. [13], with stringent measures to ensure accuracy. DNA purity was assessed using the A260/A280 ratio, and only samples with ratios between 1.8 and 2.0 were processed for PCR.

Primers targeting polymorphisms in genes related to glucose metabolism, including TCF7L2 and FTO, were designed (see Appendices for primer sequences). PCR was conducted using specific primers, and contamination prevention measures were strictly followed, with separate workstations for DNA extraction, PCR setup, and post-PCR procedures.

Glucose tolerance tests were administered to confirm GDM diagnoses based on WHO criteria. Additionally, data on participants’ awareness of GDM, access to health education, and participation in public health programs were collected to assess the effectiveness of current public health strategies.

Public health strategy analysis

To assess public health strategies, the study evaluated participants’ awareness of GDM risk factors, availability and use of prenatal care services, and engagement in community health education programs. This component aimed to identify gaps in current public health approaches and recommend strategies to enhance early detection, prevention, and management of GDM through community-based interventions and genetic screening initiatives.

Statistical analysis

Data were analyzed using IBM SPSS Statistics for Windows, version 27 (IBM Corp., Armonk, NY). Descriptive statistics summarized participant characteristics, while chi-square tests evaluated associations between categorical variables. Logistic regression analysis assessed the impact of genetic markers, lifestyle factors, and public health strategies on GDM risk, with significance at p < 0.05.

Ethical statement

Institutional Review Board of Khyber Teaching Hospital-MTI KTH issued approval 946/DGO/KMC. Consent was obtained by all participants in this study. All participants were provided with detailed information about the study’s objectives, procedures, potential risks, and benefits, and informed consent was obtained from each participant prior to their inclusion in the study. The study adhered to the ethical guidelines outlined in the Declaration of Helsinki, ensuring respect for participants’ rights, privacy, and confidentiality throughout the research process.

## Results

The research included a total of 260 individuals, with an average age of 28.5 ± 5.2 years (Table 1). The participants’ ages spanned the following ranges. The age distribution of the 18-24 age group is as follows: 64 individuals (24.62%) are 24 years old, 141 individuals (54.23%) are 23 years old, and 55 individuals (21.15%) are 22 years old. Regarding educational achievement, 25 individuals (9.62%) were unable to read or write, 41 (15.77%) had finished high school, 82 (31.54%) had completed college, and 112 (43.08%) had finished university. The proportion of parity was virtually identical, with 133 individuals (51.15%) being multiparous and 127 people (48.85%) being multiparous. The individuals’ levels of physical activity varied: 97 (37.31%) were inactive, 131 (50.38%) were moderately active, and 32 (12.31%) were active. The analysis of dietary habits indicated that 131 individuals (50.38%) adhered to a well-balanced diet, while 129 participants (49.62%) had a high intake of sugar.

**Table 1:** Demographic, lifestyle, and genetic characteristics of GDM participants (n = 260) GDM, gestational diabetes mellitus

Characteristic		Distribution
Age group in years, n (%)	18-24	64 (24.62)
	25-34	141 (54.23)
	35-44	55 (21.15)
	Mean ± SD	28.5 ± 5.2
Education level, n (%)	High school	41 (15.77)
	College	82 (31.54)
	University	112 (43.08)
	Illiterate	25 (9.62)
Parity, n (%)	Nulliparous	127 (48.85)
	Multiparous	133 (51.15)
Physical activity, n (%)	Sedentary	97 (37.31)
	Moderate	131 (50.38)
	Active	32 (12.31)
Dietary habits, n (%)	High sugar intake	129 (49.62)
	Balanced diet	131 (50.38)
Genetic markers, n (%)	TCF7L2 risk variant	52 (20.00)
	FTO risk variant	73 (28.08)

The study included 260 individuals diagnosed with gestational diabetes. The participants’ clinical and anthropometric data were analyzed, and it was found that they had a mean BMI of 27.1 ± 4.8, as shown in Table 2. Out of the total participants, nine individuals had a body weight below the normal range (3.46%), 68 had a body weight within the normal range (26.15%), 106 were over the normal weight range (40.77%), and 77 were classified as obese (29.62%). A total of 168 individuals, accounting for 64.62% of the sample, reported no family history of diabetes. In contrast, 92 participants, representing 35.38% of the sample, reported having a family history of diabetes. A total of 187 individuals, accounting for 71.92% of the sample, tested negative, whereas the remaining 73 people, representing 28.08% of the sample, were diagnosed with gestational diabetes. The mean height of the participants was 160.2 ± 6.5 cm, their mean weight was 70.4 ± 12.3 kg, and their mean waist circumference was 90.5 ± 10.2 cm. A higher proportion of GDM-positive cases carry the TCF7L2 risk variant (63.46%) and the FTO risk variant (57.53%) compared to those without these genetic risk factors.

**Table 2:** Clinical, anthropometric, and genetic risk correlation in GDM participants (n = 260) GDM, gestational diabetes mellitus

Characteristic		Distribution
BMI categories	Underweight (< 18.5)	9 (3.46%)
	Normal weight (18.5-24.9)	68 (26.15%)
	Overweight (25.0-29.9)	106 (40.77%)
	Obese (30.0 and above)	77 (29.62%)
	Mean ± SD	27.1 ± 4.8
Family history of diabetes	Yes	92 (35.38%)
	No	168 (64.62%)
Gestational diabetes diagnosis	Positive	73 (28.08%)
	Negative	187 (71.92%)
Height (cm)	Mean ± SD	160.2 ± 6.5
Weight (kg)	Mean ± SD	70.4 ± 12.3
Waist circumference (cm)	Mean ± SD	90.5 ± 10.2
TCF7L2 risk variant	Positive	33 (63.46%)
	Negative	19 (36.54%)
FTO risk variant	Positive	42 (57.53%)
	Negative	31 (42.47%)

The frequency of genetic variations in the TCF7L2 and FTO genes was examined in a group of 260 individuals diagnosed with gestational diabetes, as shown in Table 3. Out of the total participants, 52 individuals (20.00%) had the risk version of TCF7L2, whereas 208 individuals (80.00%) had the non-risk variant. In relation to the FTO gene, 187 people (71.92%) had the non-risk version, whereas 73 participants (28.08%) possessed the risk variant.

**Table 3:** Correlation of genetic variants with GDM risk and lifestyle factors (n = 260) *p < 0.05 is significant. GDM, gestational diabetes mellitus

Factor	GDM positive (n, %)	GDM negative (n, %)	Odds ratio (95% CI)	p-value
TCF7L2 risk variant	33 (63.46%)	19 (36.54%)	2.35 (1.21-4.56)	0.012*
FTO risk variant	42 (57.53%)	31 (42.47%)	1.97 (1.05-3.70)	0.045*
High sugar intake	79 (61.24%)	50 (38.76%)	2.10 (1.15-3.82)	0.017*
Sedentary lifestyle	58 (59.79%)	39 (40.21%)	1.85 (1.07-3.22)	0.025*
Engagement in public health programs	-	-	0.75 (0.58-0.97)	0.034*

Out of the individuals with the TCF7L2 risk variation, 33 tested positive and 19 tested negative for gestational diabetes, with a significant p-value of 0.012. There is a link between genetic variations and gestational diabetes. A total of 42 people were found to have gestational diabetes, whereas 31 persons tested negative for the FTO risk variation. The statistical analysis revealed a significant p-value of 0.045.

A logistic regression study that took into account genetic markers and lifestyle factors in connection to GDM risk revealed a clear correlation between the TCF7L2 risk variation and a greater risk of GDM, with an odds ratio of 2.35 (95% CI: 1.21-4.56, p = 0.012). Moreover, a strong correlation was discovered with an odds ratio of 1.97 (95% CI: 1.05-3.70, p = 0.045) between the FTO risk mutation and the probability of developing GDM. Furthermore, a significant correlation was seen between a high sugar intake and a higher risk of developing GDM, as shown by an odds ratio of 2.10 (95% CI: 1.15-3.82, p = 0.017). Moreover, leading a sedentary lifestyle increased the risk of GDM by 1.85 (95% CI: 1.07-3.22, p = 0.025), as shown in Table 3.

**Figure 1:**
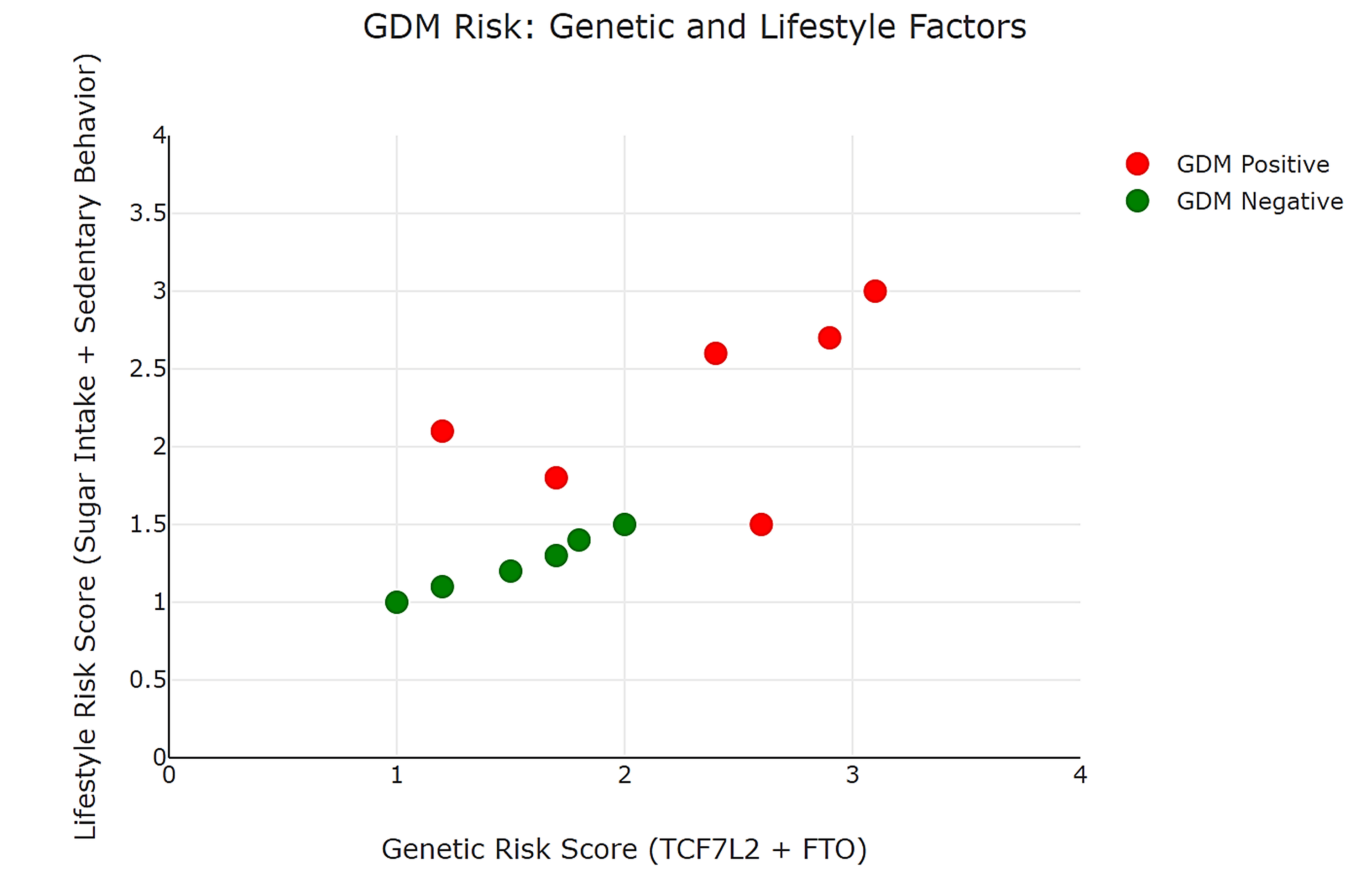
Scatter plot showing genetic variants and lifestyle factors impacting GDM risk GDM, gestational diabetes mellitus

Figure 1 shows a scatter plot that visualizes the relationship between genetic risk scores (TCF7L2 + FTO) and lifestyle risk factors (sugar intake + sedentary behavior) in GDM cases and controls. In the plot, GDM-positive cases (red markers) show higher combined risk scores for both genetics (2.4 and 2.9) and lifestyle (2.6 and 2.7), indicating a strong correlation between these factors and GDM. In contrast, GDM-negative cases (green markers) display lower risk scores in both areas (genetic scores of 1.5 and lifestyle scores of 1.2). This trend supports the hypothesis that a combination of genetic predisposition and poor lifestyle choices significantly increases the risk of developing GDM. The visualization underscores the importance of targeting both genetic screening and lifestyle interventions in public health strategies and personalized treatment for GDM.

Among the 73 people with a diagnosis of GDM, the following strategies of therapy were employed: out of the whole sample, 30 people (41.10%) used lifestyle modifications to control their condition, 25 individuals (34.25%) received insulin therapy, 10 persons (13.70%) utilized oral hypoglycemics, and eight individuals (10.96%) employed other treatment methods. The follow-up data indicate that 23 people (31.51%) continued to have GDM, whereas 50 participants (68.49%) had normal glucose levels, as shown in Table 4.

**Table 4:** Summary of treatment approaches and follow-up outcomes for GDM (n = 73) GDM, gestational diabetes mellitus

Category		Frequency (N)	Percentage (%)
Treatment approach	Lifestyle modification	30	41.10
	Insulin therapy	25	34.25
	Oral hypoglycemics	10	13.70
	Other	8	10.96
Follow-up outcome	Normal glucose levels	50	68.49
	Persistent GDM	23	31.51
Public health program engagement	Engaged	18	24.66
	Not engaged	55	75.34

The presence of genetic variants in the TCF7L2 (rs7903146, C/T) and FTO (rs9939609, A/C) genes is significantly associated with an increased risk of GDM (Table 5). Additionally, the HWE values indicate that these SNPs are in equilibrium within the studied population, further supporting the reliability of these findings in genetic screening and management strategies for GDM.

**Table 5:** Genetic variants and their association with GDM risk The p-values were calculated with logistic regression; p < 0.05 is significant. HWE values represent the p-values from the Hardy-Weinberg equilibrium tests; values greater than 0.05 suggest that the population is in HWE. SNP, single nucleotide polymorphism; GDM, gestational diabetes mellitus

Gene	SNP ID	Mutation bases	Risk variant presence	Odds ratio (95% CI)	p-value*	HWE
TCF7L2	rs7903146	C/T	Yes (20.00%, n = 52)	2.35 (1.21-4.56)	0.012	0.45
FTO	rs9939609	A/C	Yes (28.08%, n = 73)	1.97 (1.05-3.70)	0.045	0.32

## Discussion

Our study emphasizes the potential of genetic screening and public health education programs to play a pivotal role in reducing GDM rates. By integrating genetic risk assessment into prenatal care and promoting lifestyle interventions through targeted educational campaigns, public health strategies can achieve broader community health benefits. This research underscores the intricate relationship between genetic predisposition, lifestyle factors, and the development of GDM. Our findings demonstrate a strong correlation between certain genetic markers, specifically TCF7L2 and FTO, and an increased risk of GDM, alongside significant lifestyle influences such as diet and physical activity. These results provide valuable insights into the multifactorial nature of GDM and highlight areas for potential public health interventions.

Genetic factors are critical in understanding GDM. Individuals carrying the TCF7L2 risk variant exhibited an odds ratio of 2.35 (95% CI: 1.21-4.56, p = 0.012) for GDM, reaffirming the gene’s essential role in insulin signaling and glucose metabolism [14]. The TCF7L2 gene, known to influence pancreatic β-cell function and insulin secretion, plays a significant role in glucose homeostasis, making it a crucial genetic marker in the context of diabetes risk. Similarly, the FTO risk variant was associated with an odds ratio of 1.97 (95% CI: 1.05-3.70, p = 0.045), further supporting its link to both GDM and type 2 diabetes, particularly due to its associations with obesity [15]. The FTO gene, which is implicated in fat mass and obesity, highlights the genetic contributions to the obesity-diabetes nexus. Such genetic insights underscore the importance of incorporating genetic data into GDM risk assessment and management.

In light of these findings, public health strategies must leverage genetic knowledge to enhance GDM prevention and management efforts. Genetic screening programs could be integrated into routine prenatal care, facilitating early identification of high-risk individuals based on their genetic profiles. Identifying women with high-risk genetic markers, such as TCF7L2 and FTO, could enable healthcare providers to implement personalized intervention strategies, including earlier and more intensive monitoring, dietary adjustments, and tailored physical activity regimens.

In addition to genetic screening, the discussion of biotechnological advancements is paramount. Emerging biomarkers and genetic sequencing technologies can offer real-time insights into an individual’s risk profile, enhancing early detection and personalized management of GDM [5]. For instance, the utilization of advanced imaging techniques and metabolomics can help identify physiological changes associated with GDM, paving the way for more precise interventions [8]. However, the practical application of these biotechnological tools in clinical settings is still developing.

Educational programs are essential to bridge the gap between genetic risk and actionable lifestyle changes. Public health campaigns aimed at educating expectant mothers, particularly those identified as genetically at risk, could focus on maintaining a healthy diet, reducing sugar intake, and engaging in regular physical activity. Our findings indicated that sedentary behavior was associated with increased GDM risk (OR: 1.85, 95% CI: 1.07-3.22, p = 0.025), while high sugar intake posed an even greater risk (OR: 2.10, 95% CI: 1.15-3.82, p = 0.017) [16,17]. These results underscore the need for targeted interventions addressing lifestyle modification.

Such educational efforts could be integrated into community health programs, utilizing culturally sensitive approaches to reach diverse populations. Given that 54.23% of our study participants were aged 25-34 years and 35.38% had a family history of diabetes, both age and familial predisposition compound genetic and lifestyle risks for GDM. This is particularly relevant considering that individuals with a first-degree relative with diabetes face a significantly higher prevalence (14.3%) compared to those without (3.2%), with a crude odds ratio of 5 [18]. Integrating educational efforts into community health programs tailored to these high-risk groups can promote early intervention and adherence to preventive practices, ultimately reducing GDM prevalence.

Moreover, the evidence suggests that a family history not only correlates with a higher incidence of type 2 diabetes (T2D) but also that the greatest risk is seen in those with a biparental history (HR: 5.14) or a parental diagnosis before age 50 (HR: 4.69) [19]. This underscores the importance of addressing both genetic predisposition and lifestyle factors through community health initiatives, as even modest adjustments for established risk factors like BMI only slightly reduce the association between family history and T2D risk. Thus, tailored messaging and genetic counseling can empower individuals to make lifestyle changes that could mitigate their heightened risk, fostering a proactive approach to both GDM and long-term health outcomes.

Furthermore, public health policies should consider offering genetic counseling services alongside screening programs. This would provide expectant mothers with the necessary guidance on their genetic risks and advise them on evidence-based lifestyle modifications that could mitigate these risks. Counseling can be particularly beneficial in explaining the multifactorial nature of GDM, emphasizing that even with a genetic predisposition, lifestyle changes can significantly impact outcomes. Supporting this, Chan et al. [20] found that higher adherence to lifestyle modification programs (LMP) correlates with improved dietary composition and lower excessive gestational weight gain (GWG), highlighting the need for further research on how LMP can influence GDM and other maternal and infant outcomes while addressing barriers to lifestyle changes. It is crucial that healthcare systems develop robust, multidisciplinary care models integrating genetic information with lifestyle interventions. In our study, 41.10% of participants managed GDM through lifestyle modifications, while others required pharmacological interventions (34.25% insulin therapy and 13.70% oral hypoglycemics) [21]. This highlights the importance of a comprehensive management plan prioritizing non-pharmacological strategies wherever feasible while ensuring access to appropriate medications when necessary.

Study limitations and strengths

A limitation of this study is its cross-sectional design, which precludes establishing causality between genetic, lifestyle, and public health factors and the risk of GDM. Additionally, reliance on self-reported data for lifestyle factors may introduce recall bias. However, the study’s strengths lie in its diverse patient population across two major hospitals, allowing for the generalizability of findings. The inclusion of genetic analysis provides a novel perspective on GDM risk, while the focus on public health strategies offers actionable insights for improving community-based interventions and prenatal care access.

## Conclusions

The study found significant associations between the TCF7L2 and FTO genetic variants and increased GDM risk, with odds ratios of 2.35 and 1.97, respectively, alongside lifestyle factors like high sugar intake and sedentary behavior contributing to GDM prevalence. Integrating genetic screening for TCF7L2 and FTO variants into clinical practice can enhance the diagnosis and management of GDM. These genetic markers, combined with lifestyle factors, significantly impact GDM risk, suggesting that a comprehensive assessment could improve early detection and intervention strategies. Engagement in public health programs was associated with a reduced risk of GDM, highlighting the need for greater awareness and accessibility of these initiatives. Future studies should focus on larger sample sizes, long-term follow-ups to assess the progression of GDM and its complications, and the exploration of additional genetic markers or biomarkers to refine diagnosis and treatment approaches for GDM.

## Figures and Tables

**Figure 2:**
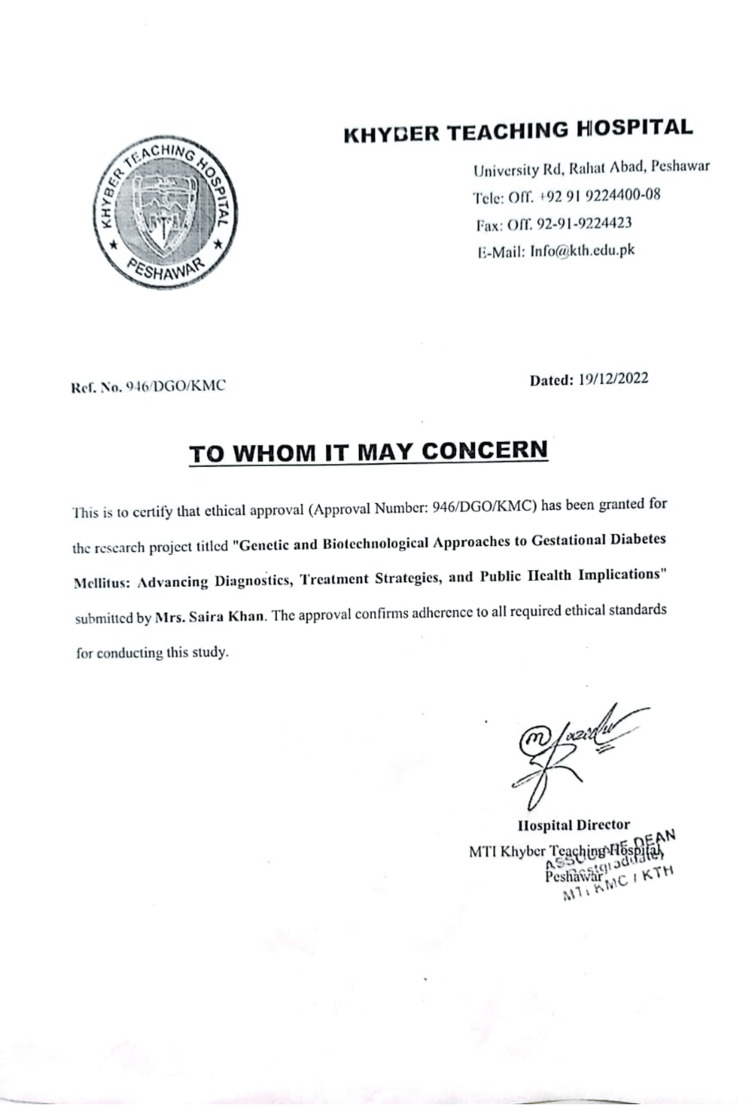
Ethical approval

## Appendices

**Table 6:** Primers for PCR amplification The PCR was performed with the following parameters: Initial denaturation: 95°C for 5 minutes. Denaturation: 95°C for 30 seconds. Annealing: TCF7L2 gene at 60°C for 30 seconds; FTO gene at 58°C for 30 seconds. Extension: 72°C for 45 seconds. Final extension: 72°C for 5 minutes. Number of cycles: 35 cycles were conducted.

Gene	Exon	Forward primer (5'-3')	Reverse primer (5'-3')	Annealing temperature (°C)	Product size (bp)
TCF7L2 gene	Exon 4	5'-AGC TGG AGT AAG TGG GGT TA-3'	5'-CTT GGA TGT CCT GTG GAG AA-3'	60	180
FTO gene	Exon 8	5'-ACA GAA TCC GCT GGA CTT AGA G-3'	5'-TGA AGT GTC TTG GAA GAA TCG-3'	58	220
